# Effect of a multidisciplinary stress treatment programme on the return to work rate for persons with work-related stress. A non-randomized controlled study from a stress clinic

**DOI:** 10.1186/1471-2458-10-658

**Published:** 2010-11-01

**Authors:** Bo Netterstrøm, Per Bech

**Affiliations:** 1 The Stress Clinic, Department of Occupational and Environmental Medicine, Bispebjerg University Hospital, DK 2400 Copenhagen, Denmark; 2 Psychiatric Research Unit, Frederiksborg General Hospital, DK-3400 Hillerød, Denmark

## Abstract

**Background:**

In recent years an increasing number of patients have been referred to the medical sector with stress symptoms. Moreover, these conditions imply increased sickness absence. This indicates a need for treatment programmes in general medical practice. The aim of this study was to test the effect of a multidisciplinary stress treatment programme on the return to work (RTW) rate in persons with work-related stress and establish predictive factors for this outcome.

**Methods:**

During a two-year period 63 out of 73 referrals to the Stress Clinic (a section of a Clinic of Occupational Medicine) completed a stress treatment programme consisted of the following:

1) Identification of relevant stressors. 2. Changing the coping strategies of the participants. 3. Evaluating/changes in participant workload and tasks. 4. Relaxation techniques. 5. Physical exercise. 6. Psychiatric evaluation when indicated by depression test score.

On average each patient attended six one-hour sessions over the course of four months.

A group of 34 employees referred to the Clinic of Occupational Medicine by their general practitioners served as a control group. Each participant had a one-hour consultation at baseline and after four months. A specialist in occupational medicine carried out all sessions.

Return To Work (RTW), defined as having a job and not being on sick leave at the census, was used as outcome measure four months after baseline, and after one and two years.

**Results:**

The level of sick leave in the stress treatment group dropped from 52% to 16% during the first four months of follow-up and remained stable. In the control group, the reduction in sick leave was significantly smaller, ranging from 48% at baseline to 27% after four months and 24% after one year. No statistically significant difference between the two groups was observed after one and two years. Age below 50 years and being a manager increased the odds ratio for RTW after one and two years, while gender and depression had no predictive value.

**Conclusions:**

The stress treatment programme showed a significant effect on the return to work rate. The stress treatment programme seems feasible for general practitioners.

**Trial Registration:**

ISRCTN04354658

## Background

In a country characterised by small to medium sized enterprises the occupational health services are not widespread and treatment of work related stress conditions is often in the hands of the general practitioners. They are however often not capable of dealing with employees suffering from work related adjustment disorders with stress symptoms and are not in the position to deal with the work places of their patients. Guidelines such as those developed in the Netherlands for managing these conditions were not available until recently [1]. To address this shortfall we established The Stress Clinic at Clinic of Occupational Medicine, Hillerød Hospital in 2002; the first of its kind in Denmark. The aim was two-fold: firstly to test a treatment concept, and secondly to use the physiological findings to increase the knowledge about the long-term effect of stress conditions. This communication describes the effect of the programme on return to work rates (RTW).

Prior to the establishment of the Stress Clinic, a visit to the Stress Reception in Stockholm [2] was undertaken in order to learn from their experiences during the previous two years. The Swedish Stress Reception treats persons referred by insurance companies with a history of long-term illness.

In addition, several published studies of the effect of intervention in persons with work-related stress were evaluated [3-9]. These studies, all from workplace settings, showed some effect of cognitive behavioural therapy and of multi-facetted interventions, but only modest effect of relaxation exercises on their own. Programmes, which focused exclusively on organisational changes had no major effect. Symptom reduction and experienced job satisfaction were the most frequently used outcome measures.

With this background, we decided that the treatment developed at the Stress Clinic should be multi-facetted, but with a simple structure so that individual elements of the full programme could also be used outside a hospital setting, e.g. in general practices with fewer available resources.

We wished to examine whether the treatment programme was able to increase RTW rate among participants compared to other stressed employees not receiving formal treatment. RTW was defined as having a job and not being absent at the time of follow-up. In addition predictive factors for RTW were explored.

## Methods

### Study design

The study was a prospective longitudinal study, in which the effect of the programme in terms of return to work in the intervention group was compared to a control group. Data was collected at baseline, after four months, after one and two years. Independent variables were participation in the programme or participation in the control group, whilst the dependent variable was return to work. Inclusion criteria were age between 18 and 60 years, attachment to the labour market (few months' unemployment acceptable), a work-related stress induced adjustment disorder, and duration ≤ 1 year. Exclusion criteria were severe medical conditions, major mental disorders such as signs of organic brain disorders, substance use disorders, schizophrenia and bipolar affective disorders.

### Material

#### The intervention group

A total of 73 persons were referred to the Stress Clinic and were given the option of participating in a four-month intervention programme, followed by evaluations after one and two years. They were treated between November 2002 and November 2004. In total 63 persons completed the treatment, although seven did not attend the one-year evaluation. Data regarding their employment-situation was collected by phone or e-mail. Almost half of the 63 people who completed the treatment had been referred by their place of work, whereas an insurance company had referred 30%, and the unemployment benefit department of their local authority had referred the rest. As for the ten Stress Clinic referrals not included in the study, in three cases the initial interview resulted in a decision that the intervention should take place elsewhere due to long-term severe depression and only two of the referrals dropped out of the treatment during the first four months. The five remaining referrals were not offered a place on the intervention programme due to lack of attachment to the labour market or primary non-occupational causes of their stress condition. For the 63 people who completed the four-month intervention programme, the average number of sessions was six. In a few cases, the treatment was extended by a couple of months.

#### The control group

The control group consisted of the 34 referrals to the Clinic of Occupational Medicine, referred by their General Practitioner during the period from 1^st ^January 2004 to 30^th ^September 2004 on the basis of a stress-related illness. They all fulfilled the inclusion and exclusion criteria met by the intervention group. The control group subjects were given the same questionnaires as the patients at the Stress Clinic, and they had two sessions with a specialist in occupational medicine, the second four months after the first. The control group subjects were contacted by post one year after their first consultation, and again after two years, in order to identify their attachment to the labour market and to collect information on their symptoms. Data was successfully collected from everyone in the control group with regard to attachment to the labour market; but only 28 completed the questionnaires (82.4%).

### The programme

We did not adhere to any specific psychological or psychiatric form of therapy. We followed the factors identified by Frank and his group [10,11] which are common across the different treatment concepts such as (a) a plausible rationale for the patients' symptoms, (b) a treatment plan, (c) a therapeutic atmosphere of a caring and hope-inspiring relationship.

The programme included:

#### The anamnesis

Before the initial interview, the participants filled out the following questionnaires:

• Basic information regarding social conditions, exercise and health

• The Stress Clinic General-wellbeing questionnaire, based on a questionnaire on stress symptoms developed by the National Institute of Occupational Health (http://www.ami.dk) and the general section of the SF-36, which deals with self-rated health domains [11]

• The WHO depression questionnaire "Major Depression Inventory" (MDI) [12,13]

The questionnaires were discussed with each participant prior to the initial interview. Based on the WHO depression questionnaire MDI, it was established whether or not the participant had a depression. Patients with an MDI score higher than 21 were referred to the Stress Clinic's psychiatric consultant. The consultant and the participant would agree on subsequent psychiatric treatment.

#### Clinical examination

Depending on the anamnesis, a clinical medical examination was carried out; supplemented by para-clinical serological tests, x-rays or further examination when indicated when indicated.

#### Stress handling sessions

During a four month period, each participant had at least four sessions, each lasting 1-2 hours, where the therapists tried to convey to the participants an understanding of stress-inducing factors, the participants' own stress-level and possible ways of reducing stress, both in relation to work and private life. The participants were given homework to do between each session, e.g. listing tasks for the next six months, prioritising planned tasks or writing down stressful events, work-related as well as private. The homework was discussed each time at the subsequent interview.

Thus the stress handling sessions had the following objectives:

1. To make the participants aware that there condition imposed a certain level of stress on their bodies. The participants were informed about the transitive nature and positive prognosis of their condition with no increased risk of setback if the treatment were followed. The participants were also informed that the treatment would take several months.

2. To diminish the strain which had caused the current poor level of functioning in the participants, e.g. through:

a. Change of workplace

b. Change of job tasks

c. Conflict resolution

d. Reduced number of working hours

e. Sick leave, possibly on a part-time basis

3. Personal social networks were involved in the process. The participants were encouraged to discuss their treatment with their spouse or other family members as well as potentially stress-reducing measures which relatives could assist with. To contribute to this engagement, the participants received their updated record after each session.

4. During the sessions, the participants acquired tools to help them handle everyday stress-inducing incidents such as traffic, children's behaviour, etc. Relaxation exercises and breathing exercises were introduced.

#### Relaxation exercises

The clinical significance of relaxation was emphasised. The participants were given a CD with a 15-minute relaxation programme, and all were encouraged to follow it every day for the duration of the treatment. The relaxation programme taught the participant to relax through concentrating on various parts of the body, one at a time, guided by the instructions on the CD. A few were also taught breathing exercises for use in panic attacks.

#### Exercise

Participants were encouraged to exercise at least twice a week. In order to evaluate the effect of the exercise, the participants' blood pressure and maximum oxygen uptake while on an exercise bike were measured at the start of the intervention and after four months. The examination was carried out at the Clinical Physiological Department at Hillerød Hospital.

#### Stress manual

The participants were given the book "Stress", published by Denmark's Radio in connection with a series of health programmes [14]. The objective was that the participants should use the book to refresh their memories regarding the information they were given during the sessions.

#### Contact with the workplace

During the intervention, the participants' place of work was contacted if adjustments to their tasks or responsibilities were needed. Such contact was only made if the participant agreed. One or more meetings would usually be held between the participant, the author of this article and the participant's supervisor in order to discuss a possible change in work allocation at the workplace. Finally, the participants were encouraged to let their work place know how they experienced their situation and the factors, which had brought it about.

The Stress Clinic target group were working people with a work-related long-term stress situation. The media were informed about the Stress Clinic and its website (http://www.stressklinik.dk), on which the treatment programme was described, together with information as to referral as well as advice about stress treatment. Treatment was paid for by the referring body, which could be an insurance company, a local authority, the person's employer, or - in one instance - the participant himself/herself. Each referral to the programme was evaluated in order to decide whether it would be appropriate to set up an initial interview. If so, a contract was sent to the referring body, and once the signed contract was received, the participant was invited to an initial interview. In some cases, further information was requested.

The initial interview lasted 1-1½ hours, and was used to assess whether there was a realistic chance that the participant would benefit from the treatment at the Stress Clinic. Inclusion criteria were labour market attachment, and stress symptoms related to working conditions. Exclusion criteria were major psychiatric disorder or other ongoing psychological or psychiatric treatment. A specialist in occupational medicine conducted this interview and the following sessions. In cases of severe depression, or where the person already participated in some kind of treatment, it was agreed with the participant that we would refer to psychiatric or other relevant treatment.

The study protocol was reviewed by the Committee System on Biomedical Research Ethics and found to be a quality development project not covered by the committee system based on the 'Guidelines about Notification of a Biomedical Research Project'. As a consequense of this informed consent was not nescesary.

#### Statistical analysis

Chi^2^-tests were used for description of the dichotomous data and t-tests for continuity data regarding baseline symptoms.

Furthermore, the odds ratio (OR) for a return to work was calculated using logistic regression analyses for the participants compared to the control group, both unadjusted and adjusted for relevant confounders: age, gender, MDI score and occupation. Finally ORs for possible predictive factors for RTW adjusted for the intervention were calculated. Statistics were calculated using SPSS, 13^th ^edition.

The level of statistical significance was P ≤ 0.05.

## Results

Table 1 shows the baseline characteristics for both the intervention group (N = 63) and for the control group (N = 34). No statistically significant differences were obtained. Approximately 50% were on sick leave in both groups and approximately 60% had a clinical depression according to MDI, but less than half of these depressed persons received antidepressive medication.

**Table 1:** Baseline characteristics for the intervention group and control group

	Intervention group	Control group
N	63	34
Women	52.4%	76.5%
Age, mean years	44.5	45.0
On sick leave	52.8%	47.8%
MDI score > 21	60.3%	61.8%
Antidepressant treatment	26.9%	22.2%
Self-rated health: Good	53.3%	55.9%
Smokers	33.0%	29.6%
Managers	17.2%	32.2%

In both the intervention group and in the control group each person had a stress condition of at least 6 months' duration. In the intervention group 43% of the referrals had experienced conflicts about role expectation at work as the major stressor, in 23% the work load (more than 60 hours per week) was the major stressor, and in 15% the major stressor was conflict with a superior at work. Six percent had experienced bullying at work and for a similar numbers the major stressor was primarily of a personal nature. These data were obtained during the treatment sessions.

**Figure 1:**
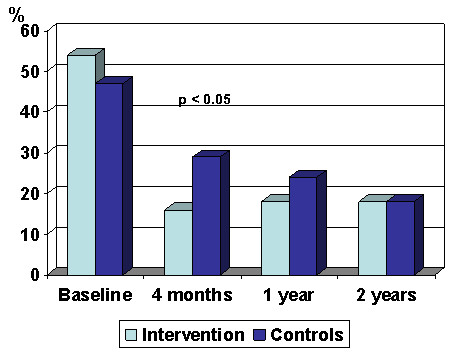
**Prevalence of participants at sick leave after 4 months, 1 and 2 years in the intervention group and the control group**.

Figure 1 shows that the number of persons on sick leave decreased linearly from 52% to 16% during the two years in the intervention group. The difference between the intervention group and the control group was statistically significant after four months. In Table 2, the odds ratio for a return to work is calculated for the intervention group, using the control group as a reference. It shows that the chance of returning to work during the first four months was 5.4 times greater in the intervention group than in the control group. Adjusting for relevant confounders changed the estimates only minimally. After one and two years, however, there were no statistically significant differences between the two groups, although with a faster return to the labour market in the intervention group. The odds ratio calculations of the importance of age, gender, level of depression and occupation for the return to the labour market among those on sick leave was as follows (Table 3): Age only became statistically significant for a return to work after one year. Gender and depression scores had no statistically significant effect, whereas being a manager at baseline increased the OR significantly after two years.

**Table 2:** Odds ratio for return to work in the intervention group compared to the control group

Follow-up time	Crude odds ratio (95% CI)	Odds ratio adjusted for age and gender (95% CI)	Odds ratio adjusted for age, gender, MDI score and occupation at baseline (95% CI)
4 months	5.4 (1.5-19.5)	5.4 (1.3-21.8)	5.1 (1.2-21.2)
1 year	2.2 (0.6-7.5)	1.3 (0.3-6.0)	1.5 (0.3-7.0)
2 years	1.7 (0.5-6.7)	0.9 (0.2-4.9)	1.2 (0.2-6.7)

**Table 3:** Participants with sick leave at baseline and odds ratio for return to work adjusted for the intervention

	OR 4 months (95% CI)	OR 1 year (95% CI)	OR 2 years (95% CI)
Age < 50 years/50+	3.1 (0.8-12.1)	17.7 (3.0-102)	38.9 (4.4-344)
Men/women	0.6 (0.2-8.0)	0.7 (0.1-1.7)	3.9(0.3-50)
Low/high depression score	1.2 (0.3-5.0)	0.5 (0.1-2.8)	0.5 (0.1-4.1)
Employment:			
White collar	0.6 (0.1-4.9)	0.8 (0.1-7.4)	15.8 (0.6-437)
Academic	1.2 (0.1-10.8)	0.3 (0.1-2.4)	6.3 (0.2-191)
Manager	0.7 (0.1-7.7)	2.8 (0.2-58.5)	60.8 (1.3-2900)
Blue collar	1	1	1

## Discussion

The results from our study indicate that:

1) It is possible to increase the rate at which people return to work through the use of elements from the stress programme

2) The stress programme has, however, no impact on attachment to the labour market in the long run (after 1-2 years).

At the one-year follow-up, the participants in the stress programme emphasised that their participation in the programme and the feeling of receiving competent support were in themselves important contributions to those activities, which they had to undertake in order to return to work. Furthermore, many of the participants said that exercising had made them feel better and had increased their level of activity. On average, their fitness as assessed by the exercise tests, increased by 10%, with those participants with the poorest level of fitness having the greatest increase. The fact that half of the participants in the stress programme had been referred by their employer also contributed to the success, because it made contact to the workplace easier, thereby paving the way for changes in working conditions.

It is problematic to use a control group in a study such as this simply because several of the control group members received other kinds of treatment during the follow-up period. The Stress Reception in Stockholm has had the same experience [2]. This means that the effect of the present stress programme must be evaluated in relation to other types of treatment involving psychologists and psychiatrists.

The main weakness of the study was however that it was not possible to recruit participants from the same source. This might affect the results in favour of the intervention group. However the MDI scores and self rated health at baseline were approximately the same in the two groups, which indicates that the comparison is acceptable

The time spent on study sessions was between four and eight hours per participant, and that is somewhat more than patients can expect to get from their GPs. For general practise setting our recommendation would be four to six 30-minute sessions over a three to four months' period when dealing with a severely stressed patient. Within this time frame, it is possible to use most of the stress treatment tools covered by this programme. Rather than full sick leave, only part-time sick leave should be attempted, as this greatly reduces the risk of dismissal, while also making the illness less dramatic and paving the way for a return to work. Emphasis should be placed on the importance of identifying stress factors, and on the positive prognosis. It is also important to keep the stressed patient active by giving "homework" and exercises to do between the consultations. The patient who is not quite as stressed and does not need sick leave requires considerably fewer resources.

Previous studies on the effect of stress treatment programmes have, apart from those carried out by the Stress Reception in Stockholm, been based on studies implemented in the workplace setting [3-9,15-20]. Therefore these studies are qualitatively different from the present study, as by definition the contact to the workplace in these studies was much closer. The Stress Reception in Stockholm has not yet published data regarding return to work. Only one Danish study is comparable to the present study (23). This study could not demonstrate an effect on sick leave absence through conventional psychological treatment of stress conditions in an occupational medicine clinic setting.

## Conclusions

This multidisciplinary stress treatment programme showed a significant effect on the return to work rate. The programme seems feasible for general practitioners.

## Competing interests

The authors declare that they have no competing interests.

## Authors' contributions

BN: Primary investigator and main author.

PB: Mental health rating scales, psychiatric assessment.

Both authors have read and approved the final manuscript.

## Pre-publication history

The pre-publication history for this paper can be accessed here:

http://www.biomedcentral.com/1471-2458/10/658/prepub

## References

[B1] van der Klink JJ van Dijk FJ Dutch practice guidelines for managing adjustment disorders in occupational and primary health care Scand J Work Environ Health 2003 29 478 487 14712856 10.5271/sjweh.756

[B2] Perski A Rehabilitation of stress-related diseases goes on different phases and is often long-lasting (in Swedish)] Läkartidningen 2004 101 14 1292 1294 15101217

[B3] Hurrell JJ Jr Murphy LR Occupational stress intervention Am J Ind Med 1996 29 4 338 341 10.1002/(SICI)1097-0274(199604)29:4<338::AID-AJIM11>3.0.CO;2-2 8728135

[B4] Murphy LR Stress management in work settings: a critical review of the health effects Am J Health Promot 1996 11 2 112 135 10163598 10.4278/0890-1171-11.2.112

[B5] Bellarosa C Chen PY The effectiveness and practicality of occupational stress treatment interventions: a survey of subject matter expert opinions J Occup Health Psychol 1997 2 3 247 262 10.1037/1076-8998.2.3.247 9552295

[B6] van der Hek H Plomp HN Occupational stress treatment programmes: a practical overview of published effect studies Occup Med 1997 47 3 133 141 10.1093/occmed/47.3.133 9156467

[B7] Reynolds S Interventions: what works. what doesn't? Occup Med 2000 50 315 319 10.1093/occmed/50.5.315 10975127

[B8] Edwards D Burnard P A systematic review of stress and stress treatment interventions for mental health nurses J Adv Nurs 2003 42 2 169 200 10.1046/j.1365-2648.2003.02600.x 12670386

[B9] Arthur AR When stress is mental illness: A study of anxiety and depression in employees who use occupational stress counselling schemes Stress and Health 2005 21 273 280 10.1002/smi.1069

[B10] Frank JD Persuasion and Healing 1961 Second rev Baltimore: The Johns Hopkins University Press 1973

[B11] Becker N Bondegaard Thomsen A Olsen AK Sjøgren P Bech P Eriksen J Pain epidemiology and health related quality of life in chronic non-malignant pain patients referred to a Danish multidisciplinary pain center Pain 1997 73 393 400 10.1016/S0304-3959(97)00126-7 9469530

[B12] Bech P Rasmussen NA Olsen LR Noerholm V Abildgaard W The sensitivity and specificity of the Major Depression Inventory. using the Present State Examination as the index of diagnostic validity J Affect Disord 2001 66 2-3 159 164 10.1016/S0165-0327(00)00309-8 11578668

[B13] Olsen LR Jensen DV Noerholm V Martiny K Bech P The internal and external validity of the Major Depression Inventory in measuring severity of depressive states Psychol Med 2003 33 351 356 10.1017/S0033291702006724 12622314

[B14] Netterstrøm B Stress (in Danish) 2002 DR Multimedie. København

[B15] van der Klink JJ Blonk RWB Schene AH Dijk FHJv Reducing long term sickness absence by an activating intervention in adjustment disorders: a cluster randomised controlled design Occup Environ Med 2003 60 429 437 10.1136/oem.60.6.429 12771395 PMC1740545

[B16] Blonk RWB Brenninkmeijer V Lagerveld SE Houtman ILD Return to work: A comparison of two cognitive behavioural interventions in cases of work-related psychological complaints among the self-employed Work and Stress 2006 20 129 144 10.1080/02678370600856615

[B17] Brouwers EPM Terluin B Tiemens BG Verhaak PFM Patients with Minor Mental Disorders Leading to Sickness Absence: A Feasibility Study for Social Workers' Participation in a Treatment Programme British Journal of Social Work 2006 36 127 138 10.1093/bjsw/bch229

[B18] Brouwers EPM Tiemens BG Terluin B Verhaak PFM Effectiveness of an intervention to reduce sickness absence in patients with emotional distress or minor mental disorders: A randomized controlled effectiveness trial General Hospital Psychiatry 2006 28 223 229 10.1016/j.genhosppsych.2006.02.005 16675365

[B19] Rebergen DS Bruinvels DJ van der Beek AJ van Mechelen W Design of a randomized controlled trial on the effects of counseling of mental health problems by occupational physicians on return to work: the CO-OP-study BMC Public Health 2007 7 183 10.1186/1471-2458-7-183 17655758 PMC1976112

[B20] Lander F Fiche C Tornemand H Andersen JH Kirkeskov L Can we enhance the ability to return to work among workers with stress-related disorders? BMC Pub Health 2009 9 372 376 10.1186/1471-2458-9-372 PMC2765963 19804632

